# A Low‐Cost and Compact High‐Frequency Gallium Nitride Gradient Power Amplifier for Low‐Field MRI

**DOI:** 10.1002/mrm.70221

**Published:** 2025-12-12

**Authors:** N. Reid Bolding, Jacob Hannan, Christopher Vaughn, Aria Patel, Snow Lin, Jessie E. P. Sun, William Grissom, Mark A. Griswold

**Affiliations:** ^1^ Department of Physics Case Western Reserve University Cleveland Ohio USA; ^2^ Department of Radiology University Hospitals of Cleveland Cleveland Ohio USA; ^3^ Department of Biomedical Engineering Case Western Reserve University Cleveland Ohio USA

**Keywords:** accessibility, gradient power amplifier, hardware, low‐field

## Abstract

**Purpose:**

To reduce the upfront cost of small, low‐field MRI systems, while expanding the capabilities of their gradient systems.

**Methods:**

A gradient power amplifier was designed to leverage the lowering cost of Gallium Nitride (GaN) power transistors and high speed logic, to achieve high efficiency and responsiveness for driving gradient coils. The switching H‐bridge design was realized as a prototype and tested to determine power output capabilities. With a digital control system, the prototype was further tested using a load which simulates a small gradient, such as those used in head and extremity low‐field MRI systems. Additionally in this test, the noise spectra produced in operation are analyzed.

**Results:**

The amplifier combined with an example control system to drive 15 A into a 225μH, 0.3Ω load simulating an effective strength over 15 mT/m and slew over 32 T/m/s, has a total build cost of under US$300 and an amplifier size under 6×6×2cm. High efficiency allows for this performance with no active cooling at full duty cycle, and high frequency switching produces controllable interference when imaging frequencies lay in the same range.

**Conclusion:**

Using GaN transistors, a low‐cost gradient amplifier can be implemented that will reduce the cost and size of low‐field MRI systems, improving accessibility.

## 1 Introduction

A major barrier to democratizing MRI in low resource settings is the upfront cost of MRI systems. In conventional systems, the power amplifiers which drive the gradient coils have been estimated to account for 15% of the total upfront system cost [1]. Even when the primary cost, that of the magnet and cryostat, is reduced by using a lower field from permanent magnet arrays, off‐the‐shelf gradient power amplifiers (GPAs) are often used, and cost thousands of US dollars. With reduced magnet cost, these can dominate the overall cost of a low‐field MRI scanner.

A more efficient and compact amplifier design using faster, lower‐loss switching elements has the potential to reduce the cost and size of this system significantly. While common in commercial high‐field MRI systems, most inexpensive and small low‐field gradient systems have not used switching amplifier topologies [2, 3, 4, 5]. Those that have used switching topologies place a significant burden on the output filtering network to provide a sharp attenuation at the switching frequency while maintaining higher frequencies for rapid gradient operation. Due in part to heat dissipation requirements and filter sizes, most designs are large in size, which contributes to the cost and lack of portability. The AE Techron 7224 amplifier used by Cooley *et al*. [6], for example, provides a powerful 900 W output with up to 16 A or 158 V, but it weighs 20.9 kg, measures 8.9×48.3×57.8cm, and costs over US$5000 [7]. In the following, we describe the theory and design of a proposed switching GaN GPA design, and the construction and testing of a prototype, which provides adequate gradient drive power in some use cases with a fraction of the cost and size.

### 1.1 Gallium Nitride (GaN) Power Transistors

Here, we take advantage of a relatively new semiconductor technology, low loss and fast‐switching Gallium Nitride (GaN) power transistors. Previous work has demonstrated the potential of GaN transistors in MRI hardware for improving RF amplifiers [8]. For gradient systems, GaN transistors allow the creation of amplifiers with higher efficiency than linear amplifiers, as well as faster switching speeds than more powerful MOSFET‐based switching amplifiers. Recent work has suggested the use of GaN amplifiers to improve gradient resolution and minimize distortion in high‐end MRI systems [9, 10]. Work by Atalar *et al*. introduced the idea of using eGaN devices to create high‐frequency GPAs [11, 12, 13]. The work presented here serves to extend these investigations and consider the use of GaN power transistors in GPAs operating at higher frequency with closed‐loop control, and demonstrate the potential for improving gradient system efficiency, size, and cost in low‐cost MRI systems.

GaN is a semiconductor with a wider bandgap than conventionally used silicon. In recent years, device designers have leveraged GaN to build power transistors, which are superior to conventional silicon power metal‐oxide semiconductor field‐effect transistors (MOSFETs) in some applications [14]. Commercially available GaN transistors have lower on resistance and higher breakdown voltages than MOSFETs, allowing for more power handling capability in smaller packages. GaN transistors require a lower charge for switching and can switch higher voltages faster than silicon MOSFETs. While newer than silicon MOSFETs, GaN transistors are becoming a well‐developed technology, and specialized drivers in small packages are commercially available at low cost to efficiently switch GaN transistors at MHz frequencies.

Silicon carbide (SiC) is another wide‐bandgap semiconductor that has shown promise in GPA power transistors [15]. When used in switching amplifiers, SiC‐based power transistors have lower spurious signals at switching frequencies than conventional silicon transistors, improving GPA resolution [9]. However, in addition to sharing many of the advantages of SiC transistors, GaN transistors are better suited for high‐frequency applications [16, 17], making them more appropriate for an amplifier with MHz‐range switching frequencies.

### 1.2 Gradient Drivers for Low‐Field MRI

In low‐field MRI systems, which target local imaging, such as a head or knee system, the gradient coils are likely smaller and lower inductance due to the reduced imaging volume. These gradient coils lower the requirements for an amplifier to provide adequate gradient slew for imaging at a given resolution. In addition, slew demands at low fields are lower due to the potential to use longer echo times [5]. For these reasons, the power amplifiers used to drive these gradient coils have reduced requirements in order to be useful for imaging. In simple approximation, for a given small gradient coil, the current the amplifier can output determines the maximum gradient strength, while the voltage determines the slew rate. To estimate the slew rate, the gradient can be considered as an ideal inductor, where the rate of current increase is given by $\frac{dI}{dt}=\frac{V}{L}$ where I is the current in the gradient coil, V is the voltage across it, and L is its inductance. With the inductance of the gradient coil in extremity imaging systems reduced, the voltage required is also reduced, but increased voltage remains beneficial for attaining higher slew rates. The system built by O'Reilly *et al*., for example, requires 10 A and 15 V for imaging with a slew rate of 30 T/m/s [5].

### 1.3 Switching Gradient Drivers

Switching GPAs, which are more efficient than linear power amplifiers, are widely used in MR systems, but only in high field MRI systems (>1T) with higher power demands. These systems use larger gradient coils to provide a larger imaging volume, which can fit more clinical use cases. These larger coils, in turn, require more power. For demanding applications like diffusion imaging, which benefit from faster and stronger gradients, even more power is required. For driving large gradients quickly and strongly, linear amplifiers, which, at best, operate at 50% efficiency, become impractical due to the large amounts of power required. To allow for high current and voltage output, where both need to be at least on the order of $10^{3}$ amps and volts, respectively, switching amplifiers have become standard [18, 19, 20]. These amplifiers are likely built from multiple large H‐bridge stages to increase power output and commonly operate at under 100 kHz [21].

### 1.4 High Frequency Switching

Usually, there is no need to operate a GPA faster than 100 kHz, with target output bandwidths around 10 kHz [19], but there are several advantages to increasing the output bandwidth and the switching frequency [11, 12, 13]. Perhaps most relevant in a cost‐optimized system is the greatly reduced switching noise in the imaging band and demand on the output filtering. With a lower power amplifier, a multistage filter with a sharp cutoff at a low frequency might take up a significant portion of the space and cost of the device. The output filter must attenuate strongly at the switching frequency to isolate switching from the rest of the system, including the feedback, transmission lines, and the gradient itself. By attenuating at the switching frequency and above, the output filter also prevents ripple or unwanted oscillations in the output due to switching. Inductors that are useful to block frequencies under 100 kHz are much larger than those that can easily block 2 MHz, and a switching frequency far away from the pass‐band demands a lower‐order filter. In some situations, increased performance as a result of larger output bandwidths may be a boon, particularly if it already comes at a lower cost.

Perhaps more importantly, a high switching frequency near the imaging RF frequency allows for steering switching noise away from imaging bands. With a switching frequency close to but outside of the imaging band, the switching harmonics are removed from the imaging band. This results in reduced switching noise in the imaging band even before the output filter.

Here, we propose a switching H‐bridge GPA based on GaN FETs and capable of switching at high frequencies. This design enables significant cost and size reduction with higher efficiency, while the high switching frequency allows for a smaller output filter, further reducing overall system size. We built and tested a proof‐of‐concept GPA to verify the suitability of the design for basic imaging in low‐field extremity MRI systems.

## 2 Methods

**FIGURE 1:**
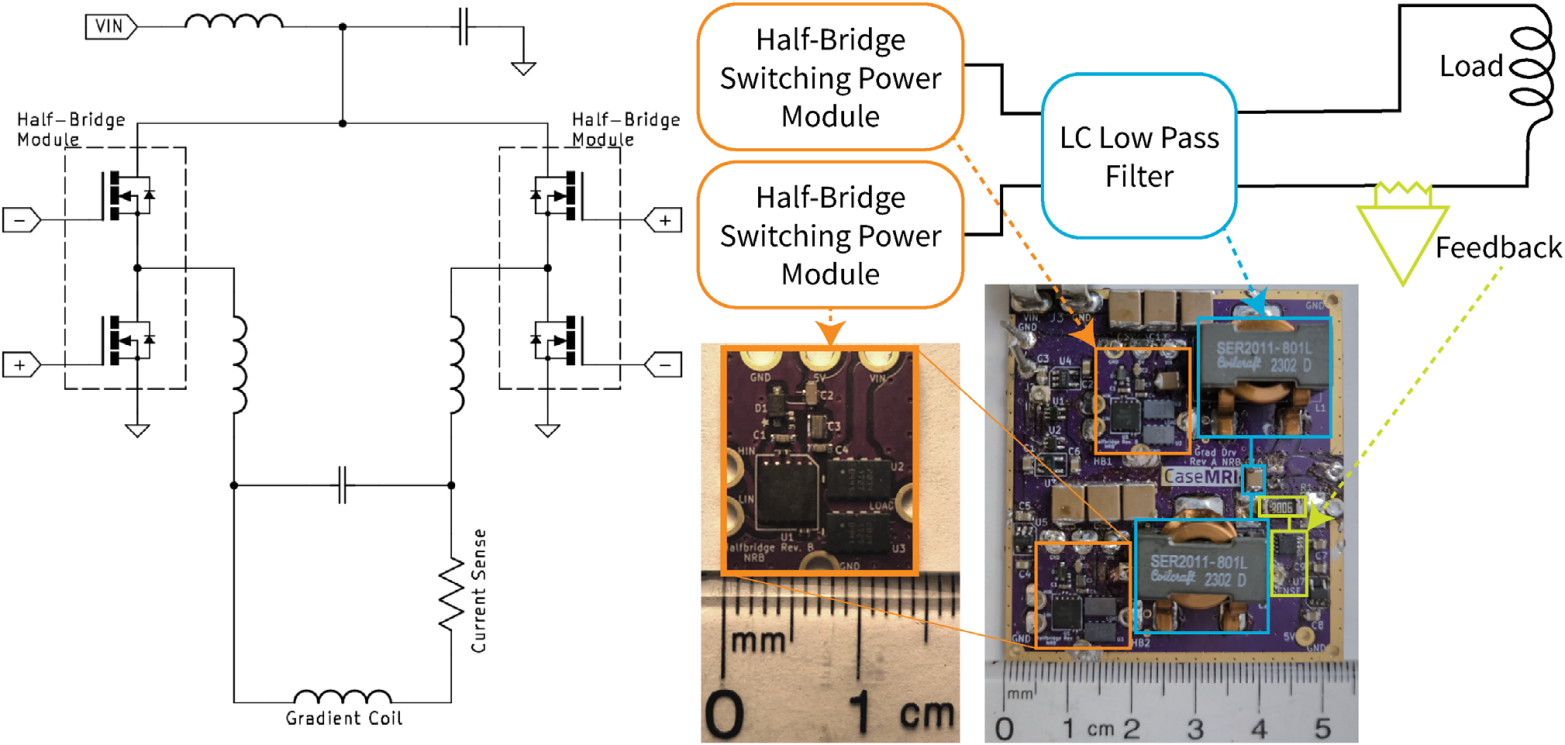
The amplifier comprises two half‐bridge modules in an H‐bridge configuration, driving the load through an LC low‐pass filter and a current sense resistor. Each half‐bridge module is driven at 2 MHz, can take in up to 100 V, and contains two eGaN transistors in a half‐bridge configuration. The footprint is 5.2×5.8cm, not including any power supply, which is not pictured. The cost to build this prototype, including parts and PCBs, was under $76.

The power conversion stage is an H‐bridge configuration of GaN transistors as seen in Figure 1. This is constructed from two half‐bridge modules, each containing two transistors, a driver, and peripherals for the driver. Modularization of the power stage allows for easier fabrication, better repairability, and parallel design iterations. This amplifier can be controlled by modulating the switching duty cycle through pulse width modulation (PWM). The amplifier itself is built onto a single compact board with control signal conditioning and feedback on the board, to allow compatibility with a range of control solutions and power sources. A current sense resistor in series with the amplifier output allows for closed‐loop control of output current.

**FIGURE 2:**
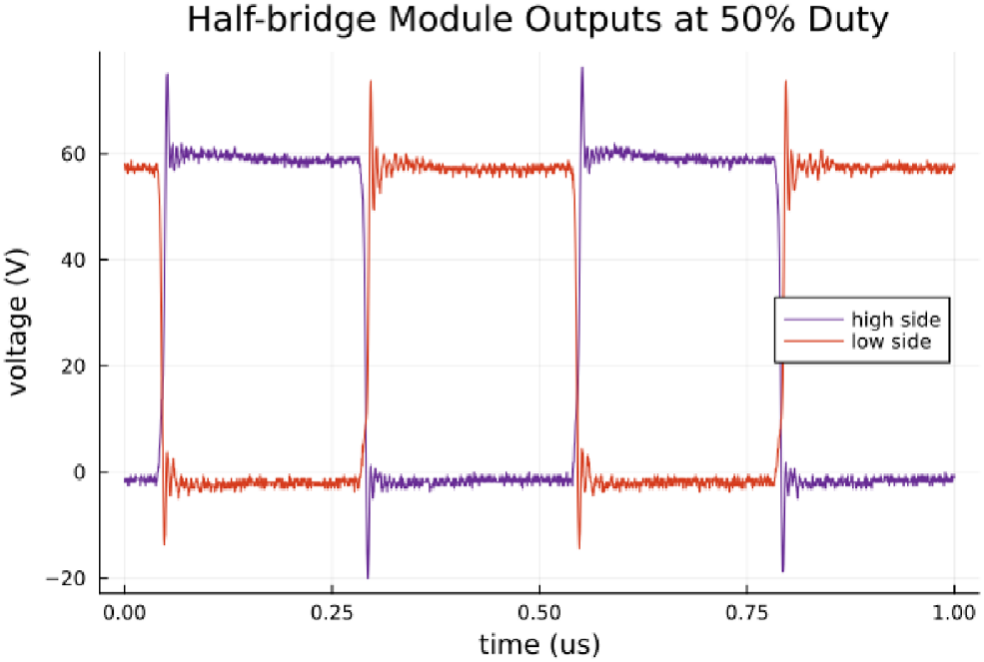
An example of H‐bridge output, with 50% duty cycle switching and 60 V bias.

The H‐bridge operates by alternating between two switching states. In the first state, current flows through the top transistor of the right half‐bridge module and through the bottom transistor of the left half‐bridge module, applying the supply voltage across the filter in one direction. This state is enabled by positively biasing the gates marked + in Figure 1. In the second state, current flows through the top transistor of the left half‐bridge module and through the bottom transistor of the right half‐bridge module, applying the supply voltage across the filter in the opposite direction. This state is enabled by positively biasing the gates marked – in Figure 1. The filter removes high‐frequency components from the square wave, effectively eliminating all frequencies above the maximum desired output frequency, including the switching frequency and harmonics. This H‐bridge topology enables the generation of any frequency within the output bandwidth, including DC. Hard switching is used to minimize conduction losses in the transistors; an example of the H‐bridge switching output is shown in Figure 2. Precision timing and high‐performance GaN transistor drivers allow hard switching with no dead‐time at these power levels. Dead‐time, time added between switching states, is often used to prevent shoot‐through, or current lost to conduction through the amplifier when states inadvertently overlap [22]. In this case, any loss to shoot‐through is low enough that a simple control system with no dead‐time is adequate.

The total material cost to build this amplifier, including parts and printed circuit boards (PCBs), is US$76 as of writing. With power supply, control, and an enclosure, the cost per channel to build a gradient driver system using this amplifier is conservatively under US$300. To use this amplifier most economically, the choice of power supply and control depends on the application.

### 2.1 Amplifier Specifications

The amplifier itself is capable of an output of ±60V and over 15 A DC, and can switch at frequencies over 2 MHz. The efficiency of the amplifier allows it to operate in these ranges at an ambient temperature near $25^{\circ }C$, without additional fans or water cooling at full duty cycle.

In typical applications, outputting frequencies beyond 100 kHz is unlikely to be necessary, so a simple output filter is adequate. The filter is composed of 800 nH ferrite core inductors and a 150 nF ceramic capacitor to block the switching frequencies before the current sense and load, blocking megahertz frequencies. This high cut‐off frequency allows the filter to be effective at switching frequencies over 1 MHz, blocking output ripple while remaining compact and capable of carrying high power. This high cut‐off frequency also provides high bandwidth control, with an output bandwidth of 150 kHz.

### 2.2 Power Supply

In most cases, the amplifier will function with a common DC power supply. To allow all stated amplifier capabilities, the supply should be able to source at least 24 V. We have found that batteries between 24 and 100 V provide low‐noise supply power, and only require a fuse and power line choke before the amplifier for safe operation.

For mains power, a transformer can provide both a voltage step‐down and galvanic isolation from the mains. The transformer in this case must be preceded by a fuse and followed by a rectifier. Depending on the number of channels required, it may be preferable to use one power supply for all channels or one smaller supply for each channel. In either case, each amplifier should be preceded by its own fuse and choke.

Our testing primarily used a benchtop DC power supply (TDK Lambda GEN60‐40 2400 W), which approximates a high‐performance battery with indefinite capacity. Similarly high performance batteries are likely to be found in solar backup systems or small electric vehicles.

### 2.3 Control

The output of this amplifier is controlled by the duty cycle of the H‐bridge switching. In particular, with an inductive load, it is necessary to control the duty cycle in a closed loop while monitoring load current. While the switching operates at 2 MHz, the output is limited to 150 kHz, so there is no need to adjust the duty cycle faster than 150,000 samples per second (150 ksps). The control system reading from the feedback amplifier is ideally at least 300 ksps to meet Nyquist sampling requirements for the whole bandwidth. For these reasons, the minimum requirements for controlling the amplifier are 2 MHz PWM, which is updated at 150 ksps, and an analog‐to‐digital converter (ADC) capable of 300 ksps.

Part of the design philosophy of this low‐cost GPA is digital‐first control. The design of the power conversion stage eliminated the explicit constant of analog synthesis in the control loop to maintain simplicity, made possible by the binary nature of the switch‐mode system. The advantage of this is implementation flexibility, in particular, the opportunity to implement fully digital control systems with binary H‐bridge control as output and digitization of the current sense feedback as input. For example, given a system with a requisite 2 MHz PWM capability at 150 ksps and ADC capability at 300 ksps, this GPA can be directly interfaced. This may be the case in low‐cost MRI systems controlled by a high‐speed micro‐controller or programmable logic device.

Many low‐cost micro‐controllers (MCUs) come close to meeting these control requirements without external components, but fall short on essential specifications, such as ADC speed or PWM precision. An MCU that meets all requirements may be difficult to source due to cost or scarcity. A less integrated control solution with discrete components may help prevent supply chain issues, in addition to remaining low cost. By adding external ADCs or external PWM circuitry to an easily sourced MCU, the gradient amplifiers presented here can be added to existing systems with little additional cost and complexity. For example, in testing, we use a Microchip PIC32CM MC00 MCU, which meets all requirements but only provides 7‐bit PWM at 1 MHz. By adding a small board to provide a saw waveform to one internal analog comparator on the chip, it is possible to use the built‐in 10‐bit, 350 ksps DAC to modulate a control waveform, which switches at 2 MHz. The switching duty cycle is set by comparing the DAC output to a sawtooth waveform at the switching frequency. Using one transistor to charge a capacitor at a constant current and a separate transistor to discharge the same capacitor, we create a circuit with a linear ramp sawtooth output voltage on the capacitor.

The feedback used for control measures the voltage across a current sense resistor in series with the load. The resistor must have high enough impedance to create a measurable differential impedance across the drive current range of the GPA, while maintaining stability and dissipating minimal power. In this case, four 2817 size 6mΩ power resistors are used in parallel. With this current sense method, one of the major limitations of this closed‐loop system is the maximum common mode voltage at the current sense feedback amplifier. In this case, the feedback amplifier we used limited the power amplifier input voltage to around 25 V. However, in order to meet basic imaging requirements, we observed that 24 V was adequate, as shown below. Furthermore, only the center 50% of the full range duty cycle (fully off to fully on) was necessary to meet specifications; as such, the amplifier was not tested beyond this range.

A complete example implementation of a digital control system for driving this H‐bridge amplifier, including firmware for the PIC32CM MC00 microcontroller, PWM generation circuits, and the feedback control algorithms, is available in the publicly accessible repository referenced in the Data Availability statement.

## 3 Results

**FIGURE 3:**
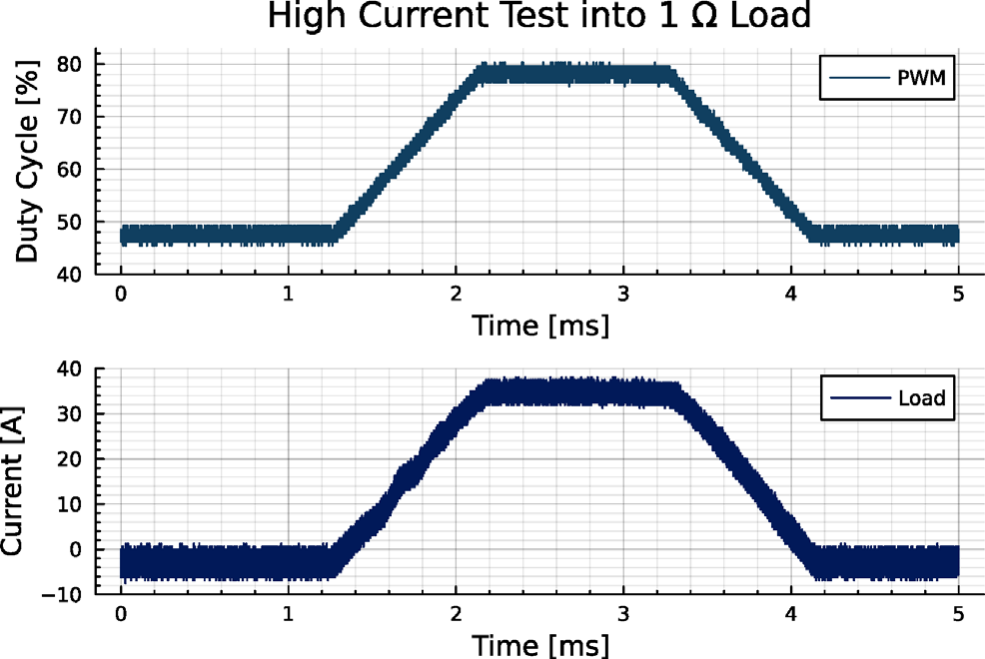
A test of the amplifier's current handling capability. A 35 A trapezoid driven into a 1Ω power resistor with no closed‐loop control. The top trace shows the duty cycle of the H‐bridge switching, and the lower trace shows the output current to the load.

We tested the power amplifier first alone to verify the operation specifications and then in conjunction with an example control system to determine practical performance. When testing without a control system, we used a 1Ω resistor as a broadband test load, as this is similar to the real impedance of many small gradient coils. Testing with this resistive load verified that the design met specified power handling capabilities and thermal performance; continuous operation at 15 A DC output with no cooling measures did not result in overheating. In this test, no active or passive cooling measures, such as the addition of a fan or heat sink, were taken. As measured by a thermal camera, the maximum temperature of the device remained stably below $100^{\circ }C$. Figure 3 shows a test of high current output where the amplifier was able to output over 30 A for one millisecond. Again, no cooling was used, but the amplifier rose to $100^{\circ }C$ during the pulse.

**FIGURE 4:**
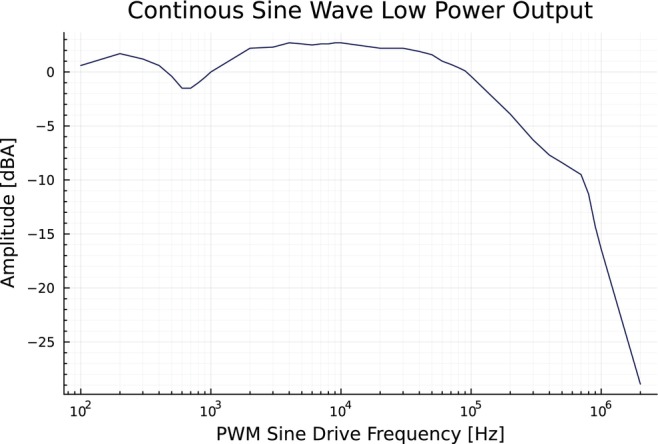
As a test of output bandwidth, we drove a continuous sine wave in PWM into a 1Ω resistor with no closed‐loop control at frequencies between 100 and 2 MHz. We measured the mean amplitude at each frequency of the current in the load with an AC current monitor and an FFT on an oscilloscope. This demonstrates a −3 dB cutoff near 150 kHz and over 27 dB of attenuation at the switching frequency of 2 MHz. An effective filter attenuates the switching frequency and harmonics strongly, but passes the full desired output band. With a high switching frequency, this simple filter is able to allow a wide output bandwidth.

We tested the half‐bridge modules and the tuning of the on‐board filters by verifying the time domain switching waveform at the half‐bridge module outputs. For efficient switching, the transition of the output between high and low levels must be sharp and involve as little ringing as possible. We additionally verified the tuning of the output filter by running PWM sine waves from a signal generator into the control input of the amplifier and sweeping the output frequency across our band of interest, DC to 2 MHz. The amplitude of the current through the load was measured with a current monitor and differential oscilloscope readings across the load. Figure 4 shows the filter cutoff starts near 150 kHz and strongly attenuates past 1 MHz, as specified, demonstrating a −3 dB cutoff near 150 kHz and over 27 dB of attenuation at the switching frequency of 2 MHz.

Output power and efficiency of the amplifier were analyzed by testing heating during DC output and finding the maximum possible output power in a trapezoid waveform.

Testing with a control system relied on an MCU, Microchip PIC32CM MC00, running a real‐time error correction while driving the PWM control of the amplifier to a target value. The error correction followed a simple algorithm, with a proportional error correction factor, which we adjusted over a serial connection to optimize performance. With this control system in place, we verified the practicality of the amplifier driving a gradient coil system, such as the one built and tested by O'Reilly et al. [5]. Using a 225μH, 0.3Ω inductor as a load to simulate such a gradient, we tested drive strength, slew rate, noise, and control linearity.

**FIGURE 5:**
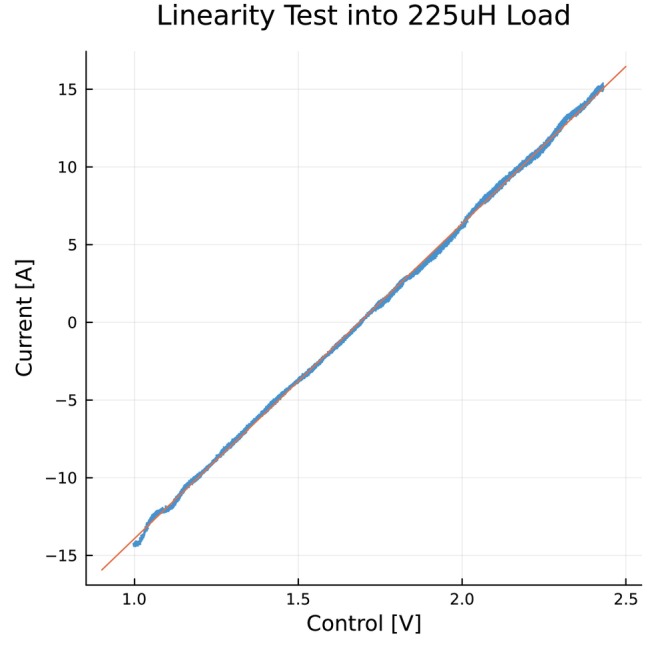
Correlation between input voltage and output current during a slow slew ramp waveform with closed‐loop control into a 225μH load shows linearity with active feedback. Plotted data are filtered to remove switching noise from measurements. A linear fit gives an $R^{2}$ of 0.9976.

Testing with the 225μH inductive load and closed loop control system showed practical performance for driving loads similar to small gradients. Figure 5 shows the control system achieved excellent linearity in a range between −15 to +15 A through the load, with a linear fit giving an $R^{2}$ of 0.9976.

**FIGURE 6:**
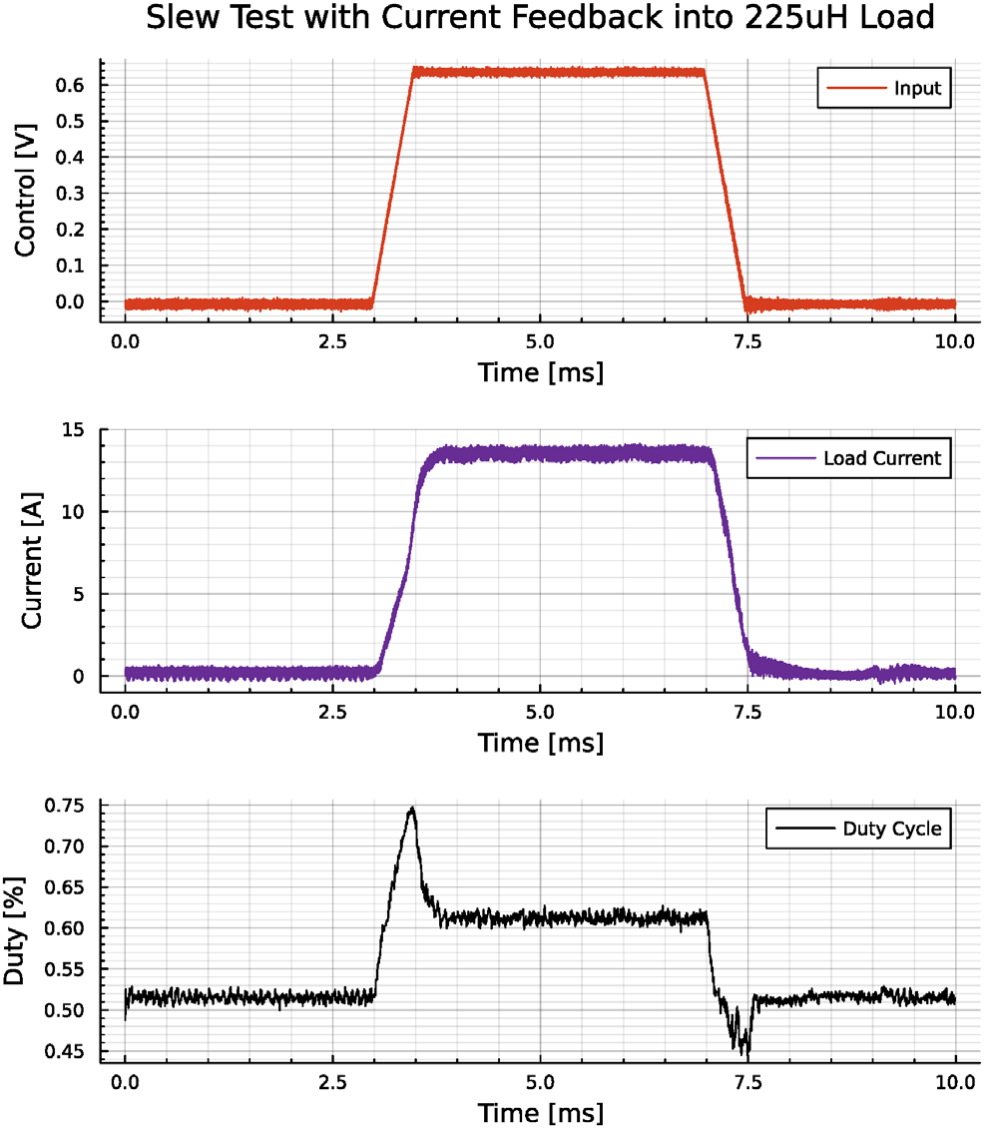
A 13 amp trapezoid waveform with a 0.4 ms rise/fall time driven with closed‐loop control shows a current slew of 32,500 A/s. For a small gradient with an efficiency of approximately 1 mT/m/A, this corresponds to a slew of 32.5 T/m/s. The top trace shows the voltage input to the control system, the middle shows the measured coil current, and the bottom shows the duty of the PWM drive.

The primary test to verify practical imaging performance was running a realistic, but relatively strong and fast, trapezoid gradient waveform in our bench‐top test setup. Figure 6 shows the driver achieved a slew rate of 32,500 A/s with a maximum value of 13 A. By monitoring heating during the strong, fast trapezoid pulse, we confirmed that the efficiency is adequate enough to not require external cooling within these performance specifications. The inductor simulates the 225μH, 0.4Ω z‐gradient coil used by O'Reilly et al. [5], which had an efficiency of 1.02 mT/m/A. This gives an effective possible drive strength of over 15 mT/m and slew of over 32 T/m/s.

**FIGURE 7:**
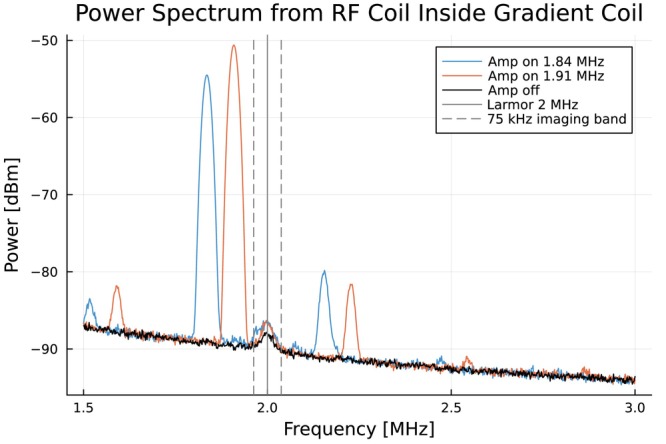
The noise from the amplifier is narrow band and high frequency. By choosing a switching frequency over 100 kHz from the Larmor frequency, noise in the imaging band can be minimized. Spectra recorded from a tuned RF surface coil placed inside the gradient load show noise levels within 2 dB of the baseline at 2 MHz when switching is set open‐loop to 1.84 or 1.91 MHz at constant frequency. The total spectral density of noise in the imaging band is 0.0009 dBm/Hz with 1.84 MHz PWM and 0.00056 dBm/Hz with 1.91 MHz PWM.

Noise measurements were performed with a spectrum analyzer measuring the signal received by a tuned 2 MHz RF coil mounted inside the gradient coil to ensure switching noise was suppressed. The 10‐turn 10 cm surface RF coil was positioned orthogonal to the B0 direction, by the inside wall of the 30.5 cm diameter gradient coil near where the leads from the gradient driver connect to the gradient coil. The noise in the load was narrow band, within 5 kHz, and isolated to the switching frequency and higher harmonics, as shown in Figure 7. Spectra recorded from the tuned RF surface coil show noise levels within 2 dB of the baseline at 2 MHz when switching is set open‐loop to 1.84 or 1.91 MHz at constant frequency. The total spectral density of noise in the imaging band is 0.0009 dBm/Hz with 1.84 MHz PWM and 0.00056 dBm/Hz with 1.91 MHz PWM.

The size of the gradient amplifier board is under 6×6×2cm, not including the power supply and control system, which may be application dependent. The cost to build the amplifier, as of writing, is US$76, including parts and PCBs. The cost of the control system used for testing is an additional US$26, as of writing, including the PIC32CM MC00 Curiosity Nano Evaluation Kit and the custom saw generator module.

## 4 Discussion

As MRI spreads into new areas of healthcare, lower cost systems may be the key to unlocking better access in resource‐restricted settings. Systems designed for accessibility can benefit from recent innovations in power systems. We show here how advanced semiconductor devices and fast logic enable high‐performance gradient amplifiers with simpler designs. Our gradient power amplifier design leverages speed and efficiency to minimize extra components, allowing smaller filters and the potential for reduced cooling systems. Possible issues with noise from switching can be mitigated by moving the switching frequency and higher order harmonics away from the imaging band, as this design is targeted at low‐cost systems operating below 10 MHz and has been tested between 1 and 2.1 MHz. The final efficiency of the amplifier is impacted by the control system. Instability and low DAC precision can quickly result in reduced amplifier efficiency, but we have verified that control from a low‐cost microcontroller is adequate for basic imaging tasks.

The output voltage is currently limited by the current sense system to around 25 V, as a common mode voltage of −25 V is too low for the current sense amplifier we used here. Current sense amplifiers capable of up to −40 V are available, and custom solutions or alternative current sense schemes may offer even better performance. Continuing work on this project will include integrating future advances in low‐cost integrated current sense solutions as they become available.

The full cost to implement our design is, of course, context dependent, but the parts and PCBs for the power amplifier itself, excluding control and power supply, cost us relatively little to build: only US$76. In systems both with an adequate control system and with existing DC supplies that already provide enough power, additional costs will also be low. We estimate a conservative material cost of US$300 per channel for this gradient driver system to be installed, including control systems, power supplies, enclosures, and the amplifier itself. This material cost is far below typical costs for a driver with similar performance, and in a form factor that is much more compact.

This low‐cost and compact design will be best applied in systems with small gradient coils, which are sensitive to the cost of the GPAs, such as systems with segmented gradients [23] or in compact and portable low‐cost MRI systems. Switch‐mode power amplifiers are able to reach higher efficiency than linear amplifiers, but are rarely employed in low‐cost MRI system designs. We present a gradient amplifier design that leverages GaN transistors to reach higher switching frequencies, thus avoiding filter size and noise concerns associated with a switch‐mode amplifier. We have verified the performance of this amplifier in a low‐cost implementation, demonstrating bidirectional current output up to 15 A at 24 V to drive a 225μH inductor. In these tests, we show that this system, at a cost of under US$300, is able to drive a small gradient for basic imaging with a 32 T/m/s slew rate. This is possible from an amplifier that is less than 6×6×2 cm and requires no external cooling. Hopefully, designs such as this can help increase the value of easier‐to‐build or procure MRI systems, allowing more people to bring MRI into new areas of patients' lives and into new parts of the world.

## Funding

This work was supported by Siemens and the National Institutes of Health (Grant No. R01EB032709).

## Disclosure

The authors' institution received research support from Siemens Healthineers.

## Conflicts of Interest

The authors declare no conflicts of interest.

## Data Availability

The code and data that support the findings of this study are openly available in Zenodo at DOI: 10.5281/zenodo.17575082. The repository includes a complete example control system design with microcontroller firmware, digital control algorithms, PWM synthesis circuits, and detailed implementation guidance for driving the H‐bridge configuration described in this work.
